# A facile system to evaluate *in vitro* drug release from dissolving microneedle arrays

**DOI:** 10.1016/j.ijpharm.2015.11.038

**Published:** 2016-01-30

**Authors:** Eneko Larrañeta, Sarah Stewart, Steven J. Fallows, Lena L. Birkhäuer, Maeliosa T.C. McCrudden, A. David Woolfson, Ryan F. Donnelly

**Affiliations:** School of Pharmacy, Queen’s University Belfast, 97 Lisburn Road, Belfast BT9 7BL, UK

**Keywords:** Microneedles, Release tests, Polymeric films, Transdermal, Quality control

## Abstract

The use of biological tissues in the *in vitro* assessments of dissolving (?) microneedle (MN) array mechanical strength and subsequent drug release profiles presents some fundamental difficulties, in part due to inherent variability of the biological tissues employed. As a result, these biological materials are not appropriate for routine used in industrial formulation development or quality control (QC) tests. In the present work a facile system using Parafilm M^®^ (PF) to test drug permeation performance using dissolving MN arrays is proposed. Dissolving MN arrays containing 196 needles (600 μm needle height) were inserted into a single layer of PF and a hermetic “pouch” was created including the array inside. The resulting system was placed in a dissolution bath and the release of model molecules was evaluated. Different MN formulations were tested using this novel setup, releasing between 40 and 180 μg of their cargos after 6 h. The proposed system is a more realistic approach for MN testing than the typical performance test described in the literature for conventional transdermal patches. Additionally, the use of PF membrane was tested either in the hermetic “pouch” and using Franz Cell methodology yielding comparable release curves. Microscopy was used in order to ascertain the insertion of the different MN arrays in the PF layer. The proposed system appears to be a good alternative to the use of Franz cells in order to compare different MN formulations. Given the increasing industrial interest in MN technology, the proposed system has potential as a standardised drug/active agent release test for quality control purposes.

## Graphical abstract

**Figure:**
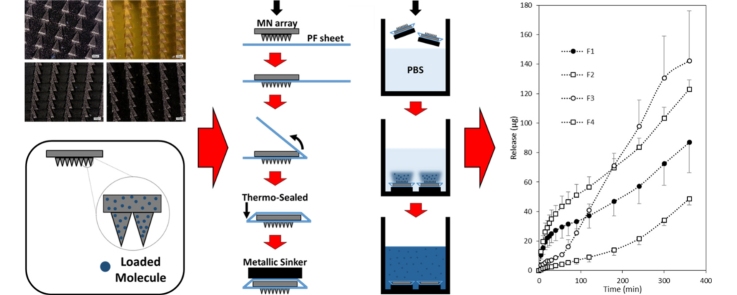

## 1 Introduction

Microneedle (MN) devices are currently attracting great interest in transdermal drug/vaccine delivery and patient monitoring (Donnelly et al., 2014a, Donnelly et al., 2012b, Mooney et al., 2014, Prausnitz, 2004, Quinn et al., 2014, Yang et al., 2013). These systems are composed of an array of micron-sized needles that painlessly, and without drawing blood, pierce and bypass the outermost layer of the skin, the *stratum corneum* (SC), which is the principal barrier to transdermal drug delivery (Benson and Watkinson, 2012, Hadgraft, 2002, Prausnitz et al., 2004). MN arrays create micro-conduits through the SC that can be used to deliver drugs to the deeper layers of the skin from where they can be absorbed directly into the systemic circulation, or to deliver vaccines to the skin-resident antigen-presenting cells (Donnelly et al., 2012b, Tuan-Mahmood et al., 2013).

MN technology is, of course, part of the broader transdermal drug delivery (TDD) area. TDD has been an important area of pharmaceutical research and development over the last four decades (Margetts and Sawyer, 2007, Prausnitz et al., 2004, Prausnitz and Langer, 2008). More recently, the market value of TDD has increased significantly from US$12.7 billion dollars in 2005 to an expected US$32 billion in 2015 (Paudel et al., 2010). However, this market is predominantly based on passive diffusion through the SC. This limits the number of molecules to only a small group (less than 20 approved drugs) that share three features: molecular mass <500 Da, relatively high lipophilicity and low required daily dose (<2 mg) (Margetts and Sawyer, 2007). As MN arrays bypass the SC, molecules delivered using this technology do not need to fulfil these requirements (Donnelly et al., 2012b). This makes MN technology an appealing approach to overcome the main limitations of conventional transdermal delivery systems while keeping its key advantages: non-invasive delivery method, avoidance of the first-pass effect, suitability for self-administration and prolonged drug release (Prausnitz and Langer, 2008). MN mediated transdermal drug delivery offers a major expansion of the route.

To date there are no MN transdermal patches on the market due to the difficulty in scale-up of fabrication (Lutton et al., 2015a). In addition, there are no currently accepted regulatory standards for MN products. The lack of MN regulation generates further difficulties regarding mass production, which requires accepted standards to assess product quality (Lutton et al., 2015a).

To date, almost all MN insertion and drug permeation studies have been carried out using biological tissue. A variety of skin models, such as heat separated epidermis, dermatomed skin, full-thickness skin, in addition to synthetic membranes have been used for these purposes (Coulman et al., 2009, Garland et al., 2012, Lee et al., 2008, Verbaan et al., 2007). Biological tissue samples are often heterogeneous, unstable, difficult to obtain and the use of biological materials sometimes presents legal issues. Therefore, these biological materials are not suitable to be routinely used in industrial formulation development or quality control (QC) tests as the tests themselves are not reproducible and, accordingly, cannot be transferred between laboratories. A good alternative to overcome these limitations in MN testing is to replace biological tissues with synthetic materials. The number of research publications detailing the use of artificial membranes in MN testing is limited however. Some examples of studies outlining the use of artificial membranes for MN insertion studies are: (Hamilton, 2011, Koelmans et al., 2013, Larrañeta et al., 2014, Muthu, 2007). Drug permeation studies using artificial membranes have been carried out by the following research teams (Donnelly et al., 2009, Garland et al., 2012, Zhang et al., 2014). Garland et al. (2012) studied the use of different skin models, including biological tissue (dermatomed and full-thickness neonatal porcine skin) and an artificial silicone membrane (Silescol^®^), to evaluate drug permeation from dissolving MN arrays. Due to the elasticity of the Silescol^®^ membrane, MN arrays did not remain inserted in the membrane but rather were withdrawn from it, thus limiting drug permeation. For this reason, Silescol^®^ membranes cannot be considered a suitable material for MN testing.

In the past, we proposed the use of Parafilm M^®^ as a model for MN insertion studies (Larrañeta et al., 2014). Continuing with the use of artificial membranes for MN characterization/testing in this work, a facile method using Parafilm M^®^ to test drug permeation using dissolving MN arrays is proposed. A series of dissolving MN arrays were prepared containing representative models of either a low molecular weight active (methylene blue) or a macromolecule (fluorescein isothiocyanate–dextran). Permeation of these molecules was evaluated *in vitro* using the proposed method and compared with conventional Franz cells permeation experiments.

## 2 Material and Methods

### 2.1 Materials

Gantrez^®^ S-97 (*M*_w_ = 1,500.000), a copolymer of methylvinylether and maleic acid polymers, was provided by Ashland (Surrey, UK). Poly(ethyleneglycol) (PEG, *M*_w_ = 10,000), poly(vinyl alcohol) (PVA, *M*_w_ = 9000–10,000, 80% hydrolyzed) and Methylene blue were obtained from Sigma–Aldrich (Dorset, UK). The isothiocyanate–dextran (FITC-dextran 70, *M*_w_ = 63,000−77,000) was obtained from TdB Consultancy AB (Uppsala, Sweden) and polyvinylpyrrolidone (PVP, *M*_w_ = 58,000) was obtained from Ashland (Surrey, UK). Parafilm^®^ M, a flexible thermoplastic sheet (127 μm thickness) made of olefin-type material was obtained from Brand GMBH (Wertheim, Germany).

### 2.2 Mehtods

#### 2.2.1 Preparation of MN arrays

Aqueous blends containing Gantrez^®^ S-97 (20% w/w), PEG 10,000 (7.5% w/w) and the selected molecules were individually used to fabricate MN arrays. Table 1 shows the formulations used in this study. This formulation was poured into laser-engineered silicone micromould templates, centrifuged for 15 min at 3500 rpm, allowed to dry under ambient conditions for 48 h. This process was followed for the preparation of dissolving MN arrays. In order to prepare hydrogel-forming MN arrays, a crosslinking step (80 °C for 24 h) was carried out after the MN arrays were dry (Donnelly et al., 2012a, Donnelly et al., 2014b). Additionally the hydrogel-forming MN arrays formulation only containing Gantrez^®^ S-97 and PEG 10,000. All the arrays (1 cm^2^) contained 14 × 14 needles with the following dimensions: 600 μm needle height and 300 μm width at the base.

#### 2.2.2 Release experiments

**Fig. 1:**
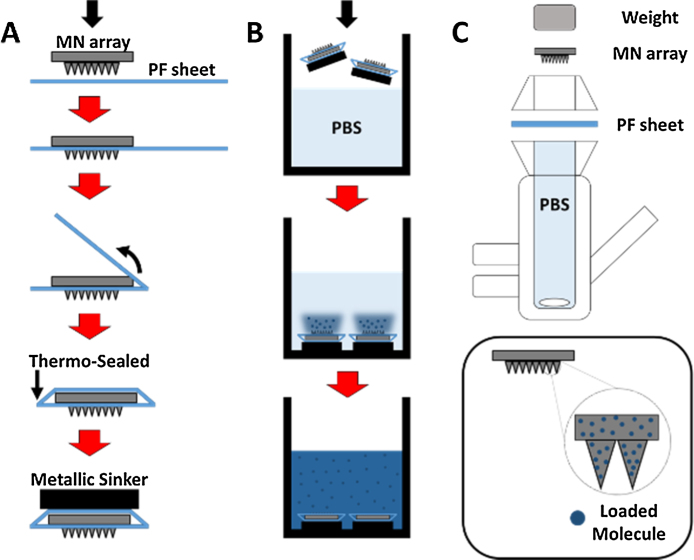
Diagrams of the insertion and preparation of the PF hermetic “pouch” (A). Diagram of the hermetic “pouch” release experiments (B). Diagram of the Franz Cell system used for the permeation experiment (C).

MN arrays were inserted in a single PF layer using a TA.XTPlus Texture Analyser (Stable Micro Systems, Surrey, UK) in compression mode (Larrañeta et al., 2014). MN arrays were placed on the surface of the PF membrane and the probe lowered onto the MN array at a speed of 0.5 mm s^−1^ until the required force was exerted (40 N). Forces were held for 30 s. Once the target force was reached, the probe was moved upwards at a speed of 0.5 mm s^−1^. PF was then folded around the baseplate of the MN array and thermally sealed, thus creating a hermetic “pouch” (Fig. 1A). A UK twenty pence coin was applied to the back part of the system as sinker (Diameter = 21.4 mm; Weight = 5.0 g; Thickness = 1.7 mm; Composition: 84% copper and 16% nickel) was applied to the back part of the system. The experiment was slightly modified for the release of hydrogel-forming MN arrays. This specific type of MN arrays was inserted in the PF and the backing layer containing MB was attached to the baseplate (Donnelly et al., 2012a). The diffusion of water will cause controlled swelling of the MN arrays creating an *in situ* hydrogel conduit. This will allow the liberation and diffusion of MB from the patch through the hydrogel MN into the release medium (Donnelly et al., 2012a). The release experiment was carried out by placing two of these closed PF/MN array systems inside a beaker containing 30 mL of phosphate buffer solution (PBS, pH 7.4) (Fig. 1B) in a thermostatic bath at 32 °C with a stirring speed of 52 strokes/min). Samples (1 mL) were extracted at defined time intervals and replaced with an equal volume of PBS.

#### 2.2.3 Franz cell permeation studies

A single layer of PF was placed on a sheet of dental wax and then a MN array was inserted into the PF using a TA.XT-plus Texture Analyser (Stable Micro Systems, Surrey, UK) as described before. The PF sheet with the MN arrays inserted was placed and secured to the donor compartment of the diffusion cell using cynoacrylate adhesive. Once MN arrays were in place, donor compartments were mounted onto the receptor compartments of the Franz cells (12 mL). A water bath system was used to heat the receptor compartment at 37 °C to bring the skin surface temperature to 32 °C (Sarmento, 2015). The patch and the MN array were kept in place during the experiment by application of a metallic weight to their upper surface. Using a long needle, samples (0.2–0.3 mL) were extracted from the receptor compartment at defined time intervals and replaced with an equal volume of receptor medium. The concentration of the selected molecule in the receiver compartment was determined using UV–vis spectroscopy. The Franz Cell system is shown in Fig. 1C.

#### 2.2.4 UV–vis and fluorescence quantification methods

Methylene blue samples were analysed using a UV–vis plate reader (PowerWave XS Microplate Spectrophotometer, Bio-Tek, Winooski, USA) at a wavelength of 664 nm.

FITC-Dextran samples were analysed using a fluorescence plate reader (BMG FLUOstar OPTIMA Microplate Reader, BMG Labtech, Ortenberg, Germany) with 493 nm and 520 nm as excitation and emission wavelengths, respectively.

Calibration curves were obtained in quintuplicate with each calibration curve contained a minimum of 8 data points. Least squares linear regression analysis and correlation analysis were performed on the obtained calibration curves (Table 2). The limit of detection (LoD) of each method was determined as follows, using Eq. (1):

(1) $$LoD=\frac{3.3\times \sigma }{S}$$

where *σ* is the standard error of the regression line and *S* is the slope of that line. Similarly, the limit of quantification (LoQ) was determined using Eq. (2):

(2) $$LoQ=\frac{10\times \sigma }{S}$$

#### 2.2.5 Microscopy and optical coherence tomography (OCT)

Microscopy images were obtained using a Leica EZ4 D digital microscope (Leica, Wetzlar, Germany) and a Keyence VHX-700F Digital Microscope equipped with a VH-Z20R lens (Keyence, Osaka, Japan). OCT images were recorder using an EX1301 OCT Microscope (Michelson Diagnostics Ltd., Kent, UK) and analysed using the imaging software ImageJ^®^ (National Institutes of Health, Bethesda, USA).

## 3 Results

**Fig. 2:**
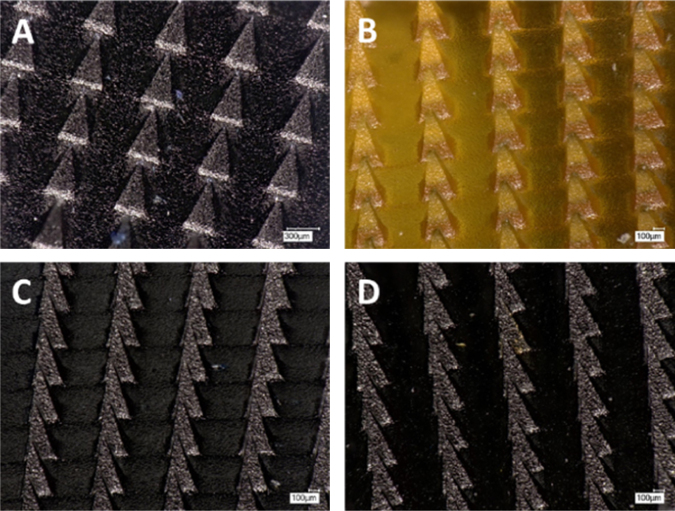
Microscopy images of 14 × 14 MN arrays made using F1 (A), F2 (B), F3 (C) and F4 (D).

**Fig. 3:**
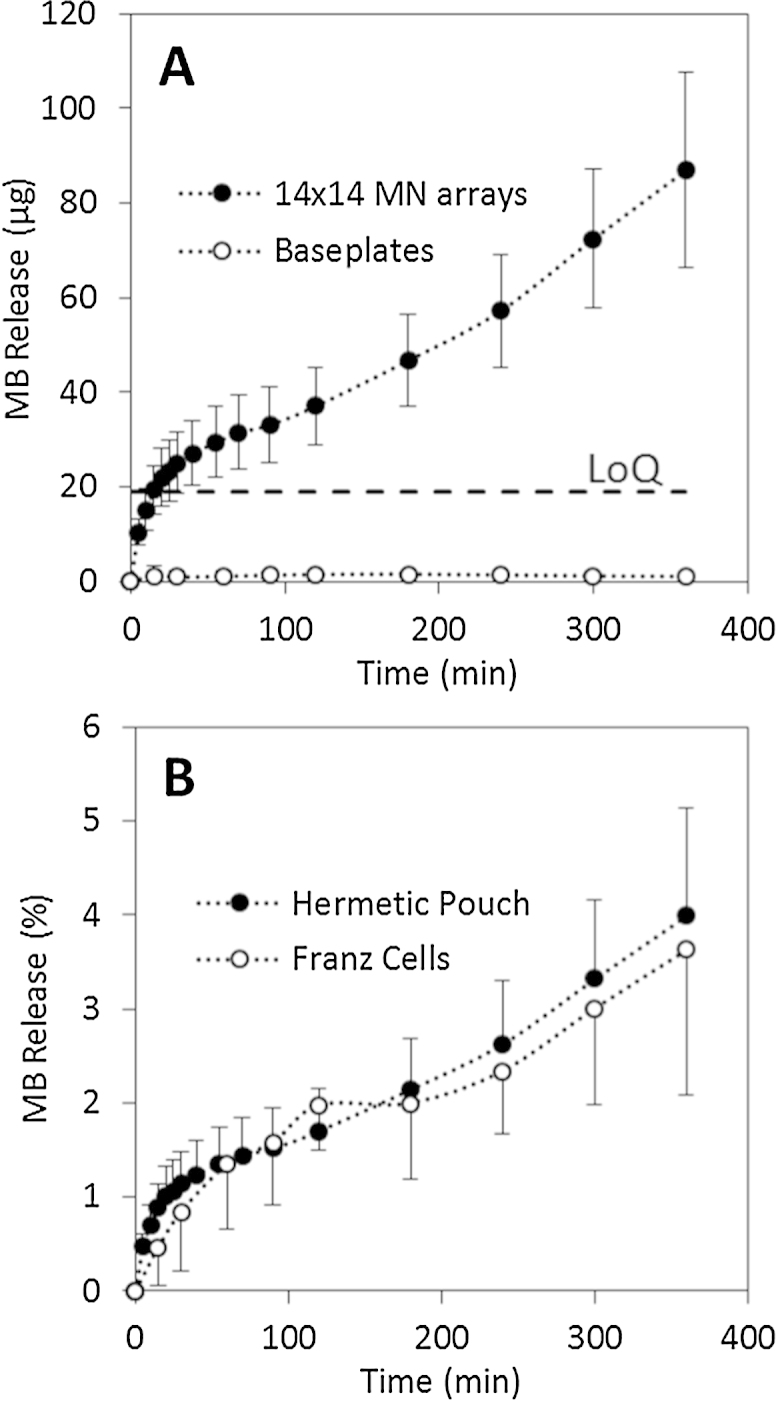
MB release from F1 14 × 14 MN arrays and baseplates through PF using the hermetic “pouch” setup (A). The dashed line shows the limit of quantification (LoQ). MB release from F1 14 × 14 MN arrays and baseplates through PF using the hermetic “pouch” and Franz cell setups (B). Means ± standard deviation, *n* = 3.

**Fig. 4:**
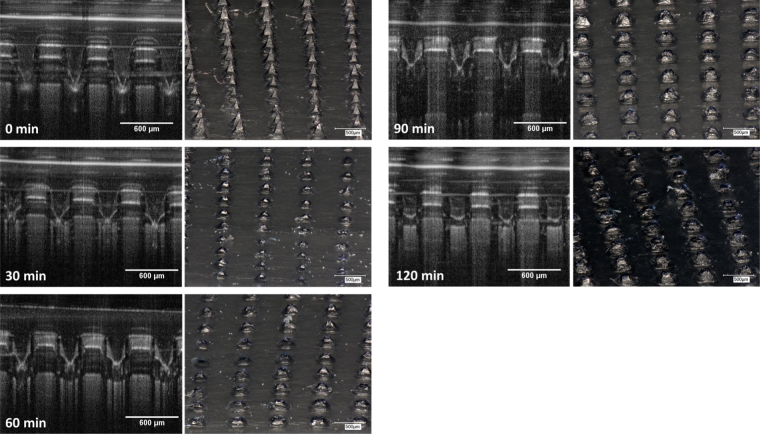
OCT (left) and microscopy (right) images of F1 14 × 14 MN arrays inserted in PF at different stages of the release experiment. Images obtained using an EX1301 OCT Microscope and a Keyence VHX-700F Digital Microscope.

Fig. 2 shows pictures of MN arrays produced using the formulations described in Table 1. All the produced arrays present consistently formed needles. Fig. 3A shows the release of MB from F1 MN arrays and baseplates using the hermetic “pouch” experimental system. Only MN arrays exhibited a release profile. This fact points out that the designed system is hermetic and only allows release from MN arrays that pierce it. The initial points of the release profile were not taken into account as the MB concentration in the release medium was below the LoQ of the system. If the release experiment was performed in a Franz cell setup, but using PF instead of biological tissue (Fig. 3B) the obtained results are similar to those obtained using the hermetic “pouch” setup. The shape of the release profiles for both experimental designs suggests that there are two steps involved in the process. Over the course of the first 30 min of experimentation, there was a burst release of MB, while after this time interval a more sustained release process took place. The first process is likely to be due to the dissolution of the needle tips that were in contact with the release medium (PBS). Once the tips were dissolved the created holes in the PF allowed the permeation of PBS inside the pouch, allowing the dissolution of the baseplates and the latter release of the MB that was loaded in them. The same phenomena was observed for the Franz Cells system and this can be explained in a similar fashion. The holes created in the PF membrane allow PBS to permeate into the baseplate surface, thus dissolving it. In order to investigate this phenomenon microscopy and OCT images of the release system were taken at different times of the release experiments (Fig. 4). In these pictures the evolution of the needle tips can be observed. It is noticeable that after 90 min the needle tip that was exposed to the release medium is totally dissolved. Furthermore, in the last pictures (120 min) it can be seen that the created holes were not closing. All these observations are consistent with the previous hypothesis. Therefore, it can be concluded that the release mechanism involves the dissolution of the needle tips and, subsequently, the permeation of the release fluid inside the “pouch”. It is noteworthy that the hermetic “pouch” approach requires the use of two MN arrays whereas those involving Franz Cell apparatus only require one MN array. In addition, the volume of release medium required for the hermetic “pouch” experimental setup was greater than that required for Franz Cell experiments. As a result and in order to obtain quantifiable drug levels, above the LoQ of the system, two MN arrays were used in this experimental design.

**Fig. 5:**
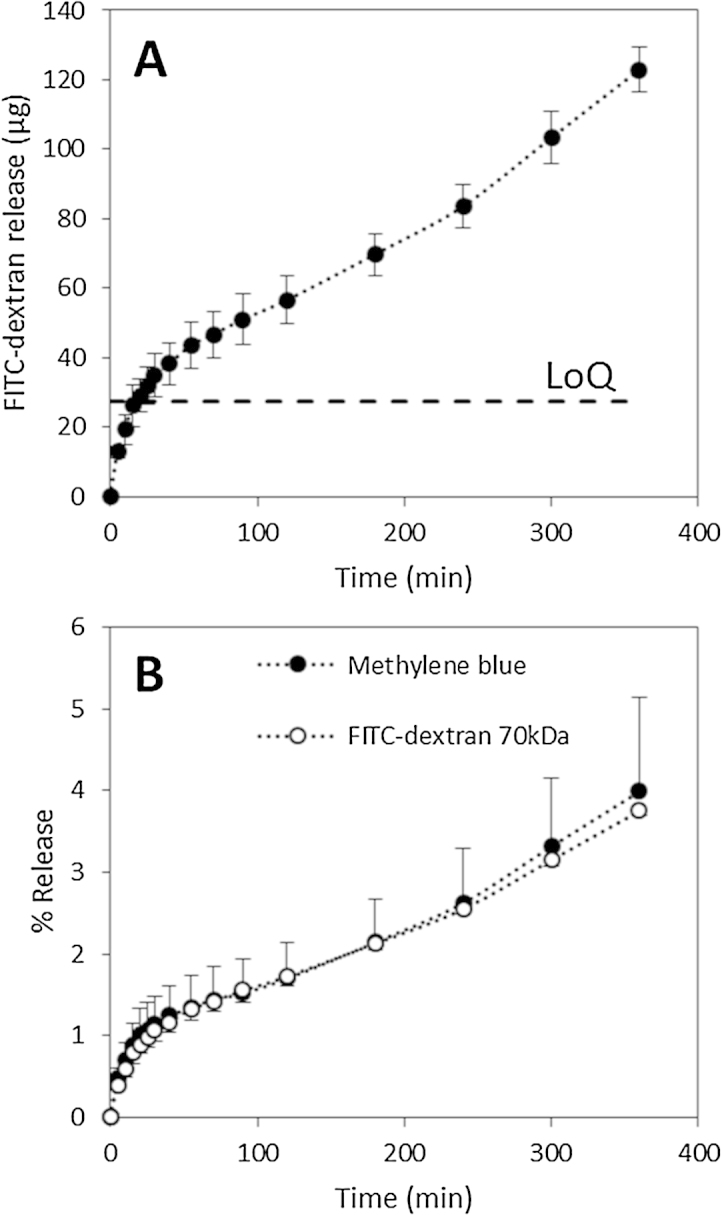
FITC-dextran release from F2 14 × 14 MN arrays through PF using the hermetic “pouch” setup (A). The dashed line shows the limit of quantification (LoQ). Comparison between FITC-dextran and MB release from F1 and F2 14 × 14 MN arrays through PF using the hermetic “pouch” setup (B). Means ± standard deviation, *n* = 3.

In addition to small molecules, the system was used to evaluate the release of a macromolecule using the same type of MN arrays. Fig. 5A shows the release of fluorescent-labelled dextran (FITC-dextran 70 kDa) using F2 MN arrays through the hermetic “pouch” release system. The release profile showed a very similar shape to those obtained for MB. Fig. 5B shows the comparison of the percentage of MB and FITC-dextran released from F1 and F2 MN arrays, respectively. The comparison was made using percentage rather than cumulative amount of the model molecule due to the different loading for both formulations (Table 1). F2 samples were prepared using a higher loading of the model molecule as the detection/quantification limits for the FITC-dextran analytical method were higher than for MB. Both curves can be considered equivalent. Similar to that observed in MB release experiments, the release of FITC-dextran over the course of the first 15 min was not taken into account when analysing experimental results as the concentration of the compound in the release medium was below the LoQ of the system.

**Fig. 6:**
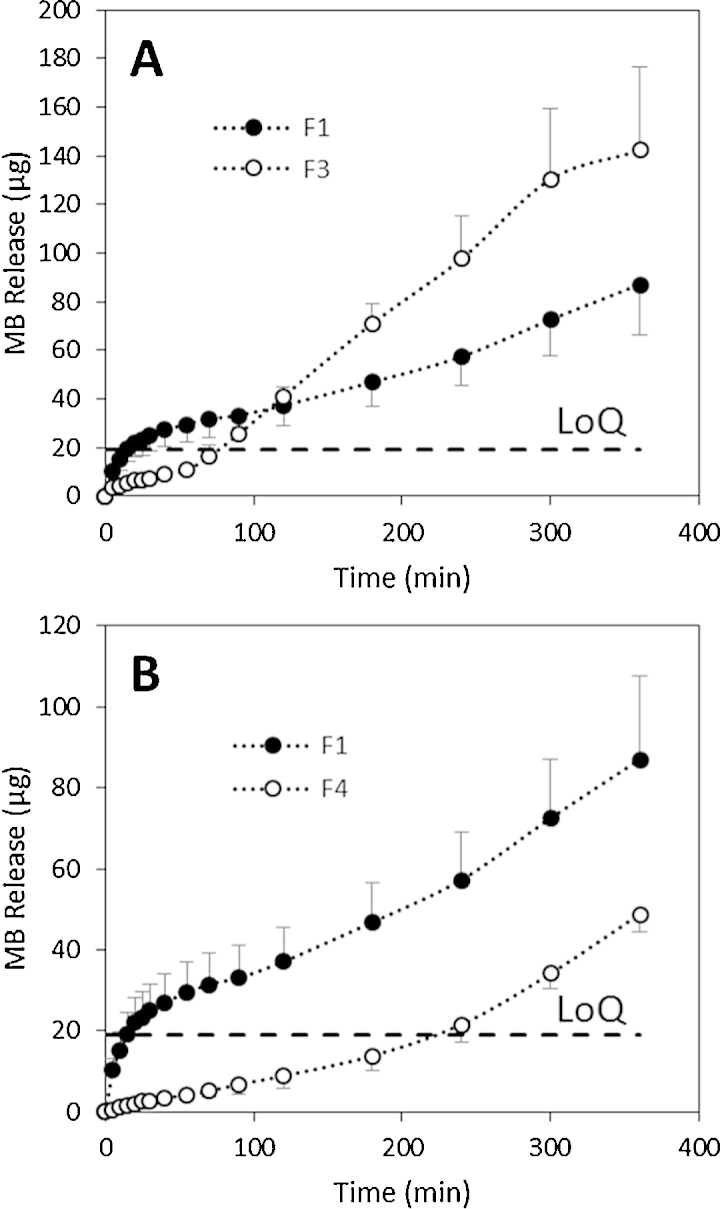
MB release from F1 and F3 dissolving 14 × 14 MN arrays through PF using the hermetic “pouch” (A). MB release from F1 and F4 dissolving 14 × 14 MN arrays through PF using the hermetic “pouch” (B). Means ± standard deviation, *n* = 3.

**Fig. 7:**
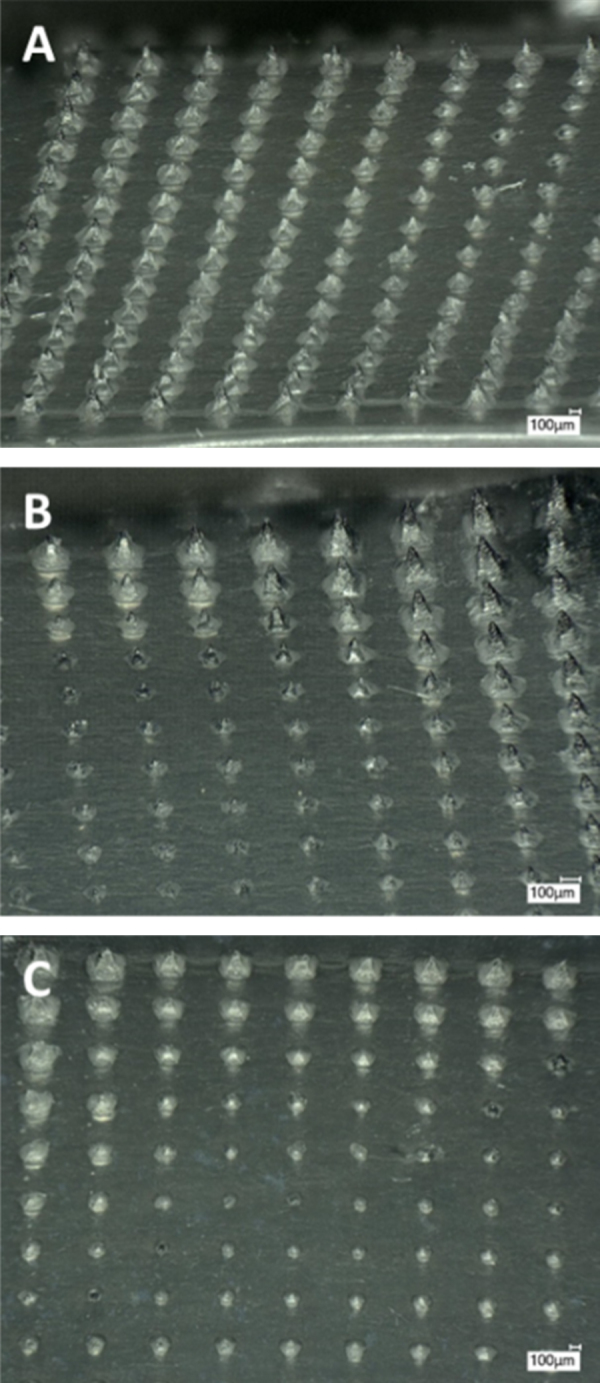
Microscopy images of F3 (A and B) and F4 (C) 14 × 14 MN arrays inserted in PF before the release experiment. Images obtained using an EX1301 OCT Microscope and a Keyence VHX-700F Digital Microscope.

The novel release system was used to evaluate the release of MB from different polymeric dissolving MN arrays. Fig. 6A shows the release of MB from F3 (PVA) and F1 (Gantrez S97 + PEG 10.000). During the first 60 min of the experiment, F3 shows a slower MB release than that of F1 but it must be noted that all MB concentrations in the release medium derived from F3 were below the LoQ of the system. After 60 min, the release of MB from F3 MN arrays accelerated. Following 90 min MB concentrations were above the LoQ and after 180 min the concentration of MB released was superior to that determined for the F1 MN arrays. The different permeation profile may be explained by the different chemical nature of the polymers. It is well known that PVA has the ability to swell partially before its dissolution (Harland et al., 1988, Quintanar-Guerrero et al., 1999). This fact could also be responsible for the slow MB release during the first hour of the experiment. Additionally, the different nature of the polymer could change the mechanical properties of the arrays, thereby affecting MN insertion. Fig. 7A and B shows the insertion of MN arrays in PF. It is noticeable that the insertion of the needles in the PF layer is not as good as for F1 MN arrays.

When a different polymer to PVP or Gantrez^®^ was used to produce MN arrays again different release profile can be observed. Fig. 6B shows the release profile of MB from F4 (PVP) MN arrays. In this case MB release was more sustained than that of F1 over the course of the experiment. Quantifiable levels of MB were only obtained after 240 min. The selected PVP present poor mechanical properties yielding MN with really brittle baseplates that can be fractured during insertion affecting MN insertion. This can be observed in Fig. 7C that shows a picture of F4 MN arrays inserted in PF. As can be seen MN pierced the polyolefin layer but the needles did not remain inserted properly.

## 4 Discussion

One of the key challenges for MN technology is the scale-up of manufacturing technologies. Nowadays, a certain number of MN-based products are being developed by different companies (Anon., 2015a, Anon., 2015b, Matriano et al., 2002, Zhang et al., 2012). However, the lack of specific quality standards and defined specifications for this novel dosage form are some of the predominant challenges facing industrial scale manufacture of MN arrays. According to the International Conference on Harmonisation: “*A specification is defined as a list of tests, references to analytical procedures, and appropriate acceptance criteria, which are numerical limits, ranges, or other criteria for the tests described”* (Lutton et al., 2015a). Therefore in order to define specifications for MNs, the basic requirements of the technology must be identified and defined. To this end, the basic requirements of MN arrays are to puncture the skin, be inserted, remain intact or dissolve (depending on the MN type) while delivering their cargo and finally to be removed intact (solid MN) or devoid of needle tips (dissolving MN) within the appropriate time scale. To the date, only a few studies evaluating some of these quality control/product specification considerations have been reported and they have mainly focused on the insertion and mechanical properties of the MN systems (Larrañeta et al., 2014, Larrañeta et al., 2015, Lutton et al., 2015a, Lutton et al., 2015b).

The *in vitro* testing of drug release is a key evaluation for the development of drug delivery and quality control systems. To the date, traditional transdermal patches can be tested following different product performance tests described by the US Pharmacopeia (Ueda et al., 2009). The simplest test consists of release testing of the transdermal patch inside a USP Apparatus following the ‘Paddle over Disk’ Method (Ueda et al., 2009). This test evaluates the release of the drug loaded in the patch from the entire surface of the patch. However, dissolving MN arrays have a different mechanism of action. The needles located in the surface of the array pierce the SC allowing the drug located in the needle to be released once the polymeric matrix is dissolved/biodegraded in the viable skin layers (Donnelly et al., 2012b, Prausnitz, 2004). Therefore, the USP product performance test does not reflect the MN mechanism of action. This is an example that highlights the need for new quality standards designed specifically for MN products.

Recently, Larrañeta et al. proposed the use of a polymeric film as a model for MN insertion studies (Larrañeta et al., 2014). In this work it was shown that Parafilm M^®^ can be used as a skin simulant for MN insertion showing good correlation with the results obtained using excised porcine skin, considered a good model for human skin (Kong and Bhargava, 2011). The present work can be considered a continuation of that study, proposing the use of the same material to design a release test that can be easily standardised as a quality control test for MN.

Initially, two different approaches were carried out using Parafilm M^®^: a Franz Cell system and a hermetic “pouch” system (Fig. 1). As can be seen in Fig. 3, both systems yield equivalent release profiles of the model molecule (methylene blue). Therefore, both appear to be valid as methods to evaluate drug release from dissolving MN arrays. Additionally, different formulations were evaluated using the hermetic “pouch” approach. This facile system was selected as it is less complex than the use of Franz Diffusion Cells. In addition to MB, the release of a larger model molecule from the dissolving MN arrays was evaluated (Fig. 5). It was noticeable that both release profiles can be considered equivalent. Therefore the release is mainly governed by the dissolution of the polymeric matrix containing the model molecules.

The release test was able to differentiate the release profiles of different formulations. Fig. 6 shows the release of PVA and PVP MN arrays loaded with MB. As explained above, different polymers will have different mechanical and chemical properties that will influence the release process. The proposed performance test is a good alternative to evaluate these differences.

In the past, artificial silicone membranes were used to evaluate drug release from MN arrays (Donnelly et al., 2009, Garland et al., 2012). In the case of Silescol^®^ membranes, the elastic nature of the membrane forced the MN arrays to retract after insertion and consequently the needle tips did not reside within the created micropores, thus yielding incomplete drug release profiles. In contrast, PF does not present the same limitation, as can be seen in Fig. 4, Fig. 7. In this case the MN tips can be seen inserted and located inside the created micropores.

**Fig. 8:**
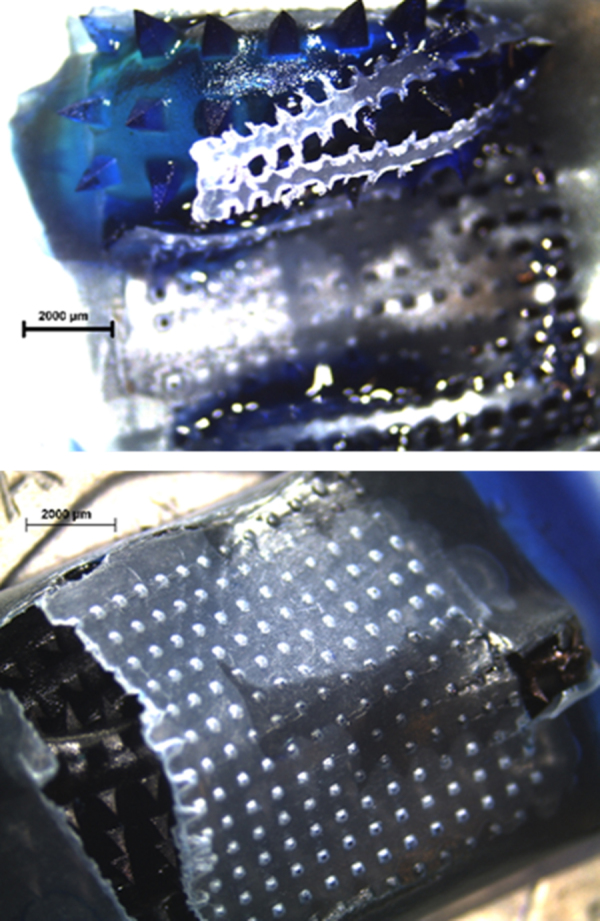
Hydrogel-forming MN arrays breaking PF hermetic “pouch” during swelling. Images obtained using a Leica EZ4 D digital microscope.

As explained above the described hermetic “pouch” system presented promising results to be used as performance test of dissolving MN arrays. We also attempted to apply the same test to hydrogel forming MN arrays. However, as can be seen in Fig. 8, this type of MN arrays could not be used in combination with the hermetic “pouch”. During the expansion process of the arrays the PF layer was broken. Hence, this method can be used mainly for dissolving and possibly coated MN arrays.

In addition to quality control, the proposed method would prove to be a good option in comparing different types of dissolving MN arrays during the formulation development phase. As pharmaceutical companies strive to shorten product development times, straightforward tests such as this could prove to be invaluable. The proposed test is capable of evaluating *in vitro* drug release from dissolving MN arrays, allowing the analyst to assess the mechanism that governs the drug release profile. Knowing the key parameters and the release mechanism of a particular drug delivery system is essential for formulation development, especially in early phases were limited amounts of drug may be available. The release of active pharmaceutical ingredients from dissolving MN arrays is mainly governed by the dissolution of the MN matrix (Donnelly et al., 2012b, Prausnitz, 2004). Using the proposed PF setup, the use of different types of polymers in the formulation of the MN arrays led to vastly different drug release profiles that could be related back to the different solubility behaviours of the employed polymers. The test therefore fulfils one of the most important requirements for this type of test in that it is capable of discriminating differences between different formulations.

To conclude, the annotated methodology is complementary to one described previously to predict MN insertion capabilities (Larrañeta et al., 2014) thus allowing the *in vitro* testing of two key aspects of MN technology for transdermal drug delivery, namely, MN insertion efficiency and drug release profile.

## 5 Conclusion

The lack of known and established product specifications is one of the main problems for MN manufacture. Universal acceptance criteria for MN specifications should be agreed by MN researchers. As MN arrays are drug delivery systems, it is clear that a release/dissolution specification should be included. Therefore, the proposed test seem to be a good alternative to cover this gap. It allows direct comparisons and, therefore, provides a quick diagnostic method to test successfully manufactured MNs. Besides, the test is facile, reliable, does not require expensive/complex equipment. This, it has the potential to test new types of MN arrays during formulation development stages. Additionally, it can complement existing techniques/protocols widely used for the physical characterization of MN arrays.

## Figures and Tables

**Table 1:** Composition of different formulations (%w/w).

Compound	F1	F2	F3	F4
Gantrez^®^ S-97	20.0	20.0	–	–
PEG 10,000	7.50	7.50	–	–
PVP 58 kDa	–	–	–	40.00
PVA 9-10 kDa	–	–	20.00	–
Methylene blue	0.50	–	0.37	0.73
FITC-Dextran 70 kDa	–	1.00	–	–
Water	72.00	71.50	–	59.27

**Table 2:** Calibration curves properties of methylene blue and FITC-Dextran as determined by linear regression and correlation analysis, LoD and LoQ.

Molecule	Slope	y-Intercept	*r*^2^	LoD (μg/ml)	LoQ (μg/ml)
Methylene blue	0.13	0.04	0.996	0.21	0.63
FITC-Dextran 70 kDa	1012.00	37.39	0.998	0.30	0.91
